# Immune checkpoint inhibitor– and phosphatidylinositol-3-kinase inhibitor–related diabetes induced by antineoplastic drugs: two case reports and a literature review

**DOI:** 10.3389/fendo.2023.1236946

**Published:** 2023-09-05

**Authors:** Yue Gao, Mingyao Zhong, Lulu Gan, Cheng Xiang, Ling Li, Yimin Yan

**Affiliations:** ^1^ Department of Endocrinology, Xiaogan Hospital Affiliated with Wuhan University of Science and Technology, The Central Hospital of Xiaogan, Xiaogan, Hubei, China; ^2^ Medical College, Wuhan University of Science and Technology, Wuhan, China

**Keywords:** immune checkpoint inhibitor, phosphatidylinositol-3-kinase inhibitor, diabetes mellitus, anti-tumor drug, cancer

## Abstract

Immune checkpoint inhibitor (ICI)- and phosphatidylinositol-3-kinase inhibitor (PI3Ki)-related diabetes mellitus are common side effects of anti-tumor drug use that present mainly as hyperglycemia. Here, we present two case reports of diabetes mellitus caused by the use of tremelimumab and apalutamide, respectively, in cancer treatment, and a comprehensive, comparative review of the literature on these forms of diabetes. Case 1 presented with diabetic ketoacidosis and was diagnosed with ICI-related diabetes mellitus and treated with insulin. Case 2 was diagnosed with PI3Ki-related diabetes mellitus, and her blood glucose level returned to normal with the use of metformin and dapagliflozin. We systematically searched the PubMed database for articles on ICI- and PI3Ki-related diabetes mellitus and characterized the differences in clinical features and treatment between these two forms of diabetes.

## Introduction

Immune checkpoint inhibitor (ICI)-related diabetes mellitus is a rare but severe side effect of the use of cytotoxic T lymphocyte–associated antigen 4 (CTLA-4), programmed cell death receptor 1 (PD-1), and programmed cell death receptor-ligand 1 (PD-L1) inhibitors, with a reported incidence of 0.2–1.27% (1). Changes in immune checkpoints lead to the abnormal proliferation and activation of T cells, which attack pancreatic beta cells, causing endogenous insulin deficiency. If this deficiency is not detected and treated with insulin in a timely manner, the risk of diabetic ketoacidosis (DKA) is high. At the time of ICI-related diabetes onset, 50–71% of patients have DKA (2).

Phosphatidylinositol-3-kinase inhibitor (PI3Ki)-related diabetes mellitus is a glucose metabolism abnormality caused by the use of PI3Kis in malignant tumor treatment, which results in PI3K-AKT–mammalian target of rapamycin (mTOR) signaling pathway blockade and insulin resistance (3). The incidence of diabetes caused by the use of apalutamide to treat hormone receptor (HR)^+^ human epidermal growth factor receptor 2 (HER2)^–^ phosphatidylinositol-4,5-bisphosphate 3-kinase catalytic subunit alpha (PIK3CA)-mutant breast cancer can reach 65% (4).

In this article, we present two cases with a comprehensive literature review and comparative analysis of these two types of diabetes caused by different types of anti-tumor drug to improve doctors’ understanding.

## Case reports

### Case 1: ICI-related diabetes mellitus

A 47-year-old man presented with dry mouth, polydipsia, polyuria, and blurred vision and was admitted to the hospital. He had been diagnosed with melanoma in the right maxilla 5.5 months previously, and had undergone one cycle of docetaxel chemotherapy followed by lesion resection and neck lymph-node dissection. He had begun to take toripalimab (a monoclonal anti–PD-1 antibody; 210 mg every 2 weeks; Shanghai Junshi Bioscience Co., Ltd., Shanghai, China) 2.5 months previously, and was on the fifth cycle of treatment. He had no history of diabetes.

**Figure 1:**
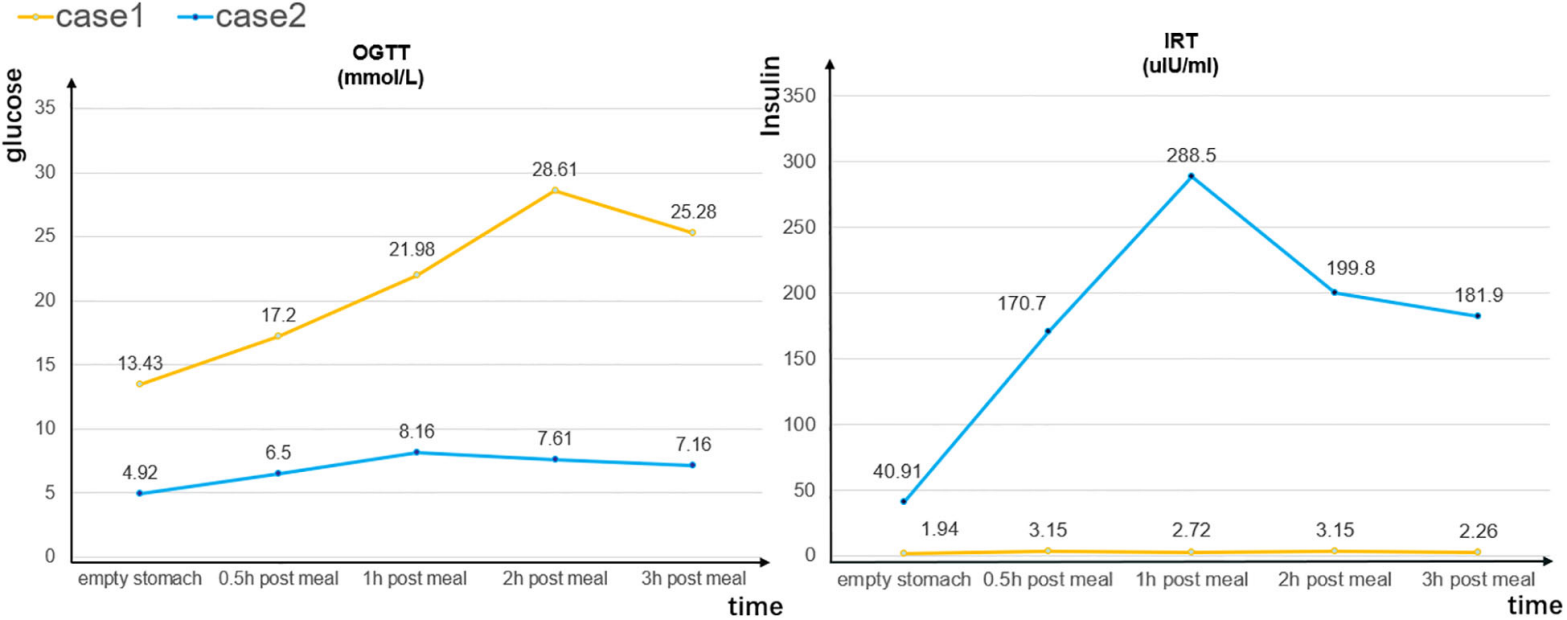
Oral glucose tolerance test (OGTT) and insulin release test (IRT) results for cases 1 and 2.

On admission, the patient’s body temperature was 36.2°C, his pulse rate was 92 beats/min, his respiratory rate was 20 breaths/min, and his blood pressure was 113/76 mmHg. Laboratory tests revealed a blood glucose level of 19.03 mmol/L (normal, <11.1 mmol/L), ketone level of 2.8 mmol/L (normal, <0.3 mmol/L), and glycosylated hemoglobin percentage of 8% (normal, <6.0%; **Table 1**). The results of an oral glucose tolerance test (OGTT), serum C-peptide measurement, and insulin release test (IRT) revealed blood glucose elevation and insufficient insulin and C- peptide secretion (**Figure 1**). Urinalysis revealed positivity for ketone bodies and strong positivity for glucose. The patient was negative for insulin-related antibodies. Electrocardiographic, chest computed tomographic, and liver, gallbladder, spleen, pancreas, and kidney ultrasonographic findings were normal, and no exudation or bleeding was observed in the fundus retina.

**Table 1:** Clinical characteristics and laboratory test results for cases 1 and 2.

Characteristic	Case 1	Case 2
Sex	Male	Female
Age (years)	47	49
Body mass index (kg/m^2^)	21.58	22.19
Neoplasia	Melanoma	Breast cancer
Treatment	Toripalimab	Alpelisib
Pretreatment history of diabetes	No	No
Time to onset (weeks)	15	2
Ketoacidosis	No	No
Fasting blood glucose (mg/dl)	342.54	208.8
Fasting C-peptide level (ng/ml)	0.6	4.6
HbA1c (%)	8	7.5
Autoantibodies	nd	nd

HbA1c, glycated hemoglobin; nd, no data.

On the first day of hospitalization, the patient was asked to stop using toripalimab. During hospitalization, he was given insulin lispro (8–10 IU subcutaneously, three times/day; Lilly Suzhou Pharmaceutical Co., Ltd., Suzhou,China) and insulin glargine (16 IU subcutaneously once a day; Sanofi Wanante Pharmaceutical Co., Ltd., Beijing, China) injections and saline infusions. At 3 days, his blood ketone level had returned to normal, but his blood glucose level remained high despite the cessation of the anti–PD-1 treatment. With continued insulin treatment, his blood glucose level had stabilized at 7 days. Considering the patient’s medical history and auxiliary examination findings, the diagnosis of ICI-related diabetes mellitus was made. At 10 days, the patient’s condition was controlled and he was discharged. He resumed the toripalimab treatment and continued the hypoglycemic treatment. On a 1-month follow-up telephone consultation, he reported that his condition was stable.

### Case 2: PI3Ki-related diabetes mellitus

A 49-year-old menopausal woman with HR^+^ HER2^–^ PIK3CA-mutant breast cancer and bone metastasis presented with 2.5 kg weight loss in the previous 2 weeks for hypoglycemic treatment adjustment. She had no dry mouth, polydipsia, polyuria, or history of diabetes. Two months previously, she had begun to take alpelisib (a PI3ki; 300 mg/day; Novartis Pharma AG, Beijing, China) as anti-tumor treatment and to monitor her blood sugar according to her doctor’s order. Her fasting and postprandial blood glucose levels had fluctuated between 6.3 and 7.3 mmol/L and between 14.7 and 27.1 mmol/L, respectively. One month previously, the patient had begun to take metformin hydrochloride tablets (0.85 g/day; Sino-American Shanghai Squibb Pharmaceutical Co., Ltd., Shanghai, China) to control her blood sugar, but without success; her maximum recorded blood glucose level was 29.3mmol/L. The patient stopped taking alpelisib on her own, and her blood glucose levels had decreased significantly. She was admitted to the hospital.

On admission, the patient’s body temperature was 36.2°C, her pulse rate was 92 beats/min, her respiratory rate was 19 breaths/min, and her blood pressure was 131/93 mmHg. Laboratory tests revealed a reduced red blood cell count (3.38 × 10^12^/L), white blood cell count (3.45 × 10^9^/L), and hemoglobin concentration (103.00 g/L), and increases in tumor markers such as carbohydrate antigen 153 (>300.00 U/mL) and carcinoembryonic antigen (17.80 ng/mL). Her blood glucose and ketone levels were 11.6 and 0.3 mmol/L, respectively, and her glycosylated hemoglobin percentage was 7.50%. The results of an OGTT and IRT showed a normal blood sugar level but hyperinsulinemia (**Figure 1**).

**Figure 2:**
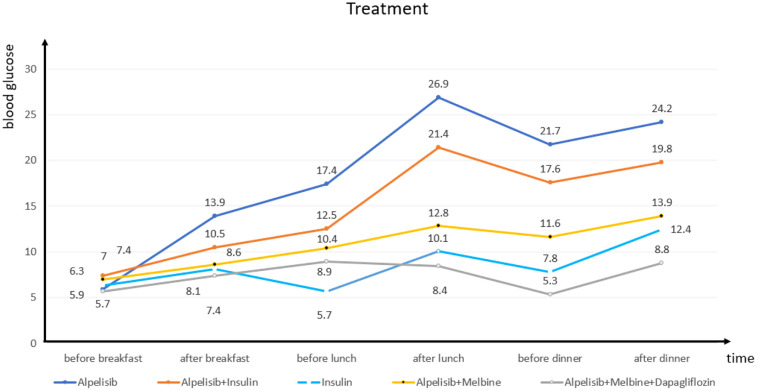
Blood glucose fluctuations during treatment in case 2.

We closely monitored the patient’s blood glucose level and found that it usually increased significantly 4 h after she had taken alpelisib and remained high (**Figure 2**). On day 3, we initiated insulin pump (Medtronic, Dublin, Ireland) treatment (insulin lispro, 56 IU/day), which did not significantly reduce the patient’s blood glucose level. On day 5, the alpelisib was stopped and the insulin dosage was halved (to 25 IU/day), but the patient’s blood glucose curve remained low. On day 8, to maintain the breast cancer treatment effect and simplify the hypoglycemic treatment, we asked the patient to resume the alpelisib and switched from the insulin pump to oral metformin (0.5 g twice a day), The patient’s blood sugar level remained below expectation. On day 12, we added dapagliflozin (10 mg once a day); for hypogly AstraZeneca Pharmaceutical Co., Ltd., Shanghai, China cemic treatment. The patient’s blood glucose level remained stable, and the diagnosis of PI3Ki-related diabetes mellitus was made. At 15 days, the patient was discharged and continued to follow the established treatment plan. One month later, she reported during a follow-up telephone consultation that her condition remained stable.

## Discussion

In recent years, ICIs and tumor-targeting PI3Kis have rapidly become new cancer treatment options, but they have been shown (1, 4) to increase the incidence of diabetes in patients using them. The forms of diabetes caused by these two types of anti-tumor drug have some similarities, but also significant differences in terms of pathogenesis, clinical manifestations, and treatment (**Table 2**).

**Table 2:** Differential diagnosis of ICI- and PI3Ki-related diabetes mellitus.

Characteristic	ICI-related diabetes	PI3Ki-related diabetes
**Incidence**	0.2–1.27%	65%
**Etiological agent**	ICI as cancer medication	PI3Ki as cancer medication
**Nosogenesis**	Insulin deficiency caused by islet β-cell damage	Insulin resistance due to PI3Ki pathway inhibition
**Clinical manifestation**	Hyperglycemia after a mean of 4.5 treatment cycles	Hyperglycemia after a mean of 2 weeks of treatment
**Laboratory findings**	Decreased insulin, C peptide levels	Increased insulin level
**Treatment**	Insulin	Hypoglycemic drugs (e.g., metformin)

ICI, immune checkpoint inhibitor; PI3Ki, phosphatidylinositol-3-kinase inhibitor.

## ICI-related diabetes

**Figure 3:**
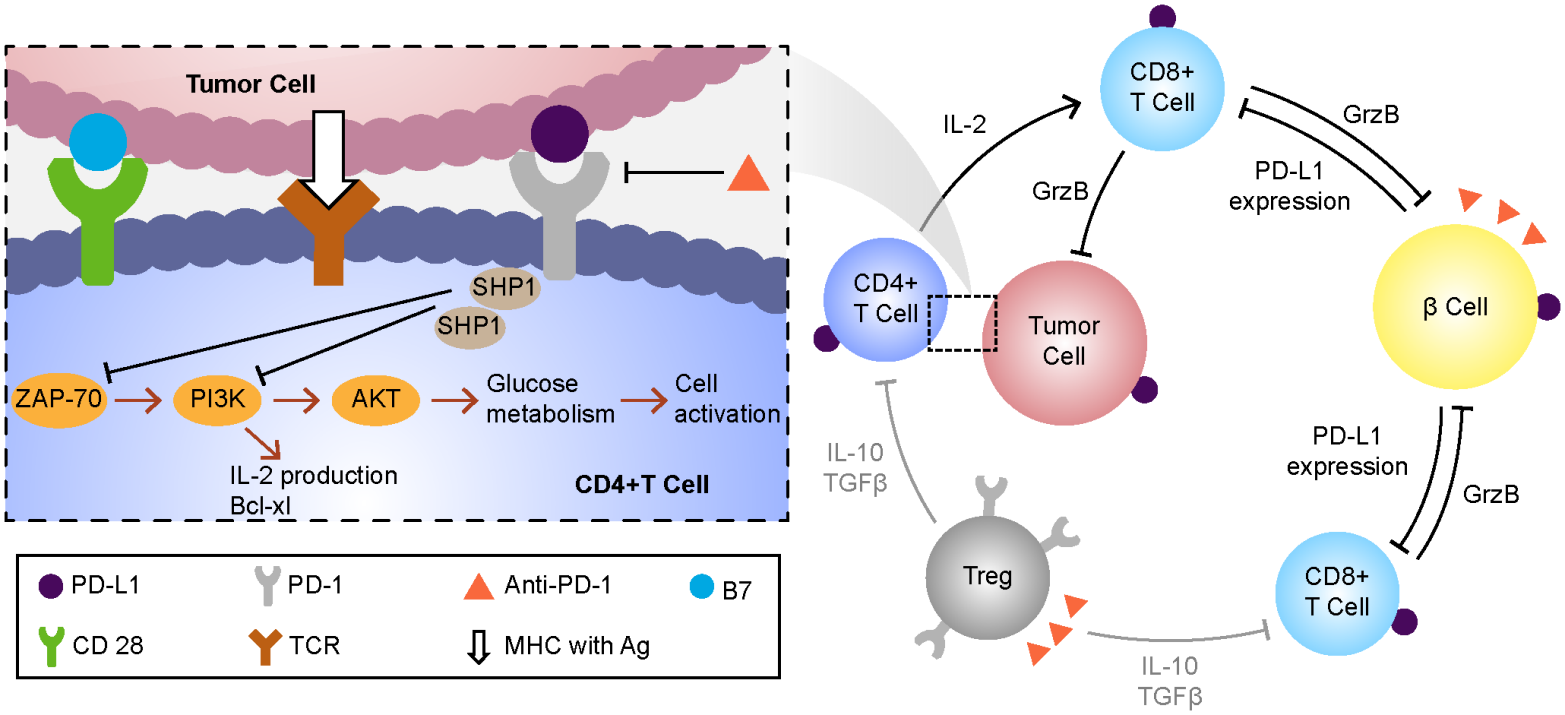
The possible mechanisms of drug action and hyperglycemia of toripalimab. MHC, major histocompatibility complex;Ag, antigen;TCR, t cell receptor;SHP-1,tyrosine phosphokinase;ZAP-70, Tyrosine protein kinase;PI3K, phosphatidylinositol -3- kinase; AKT, protein kinase B;PD1, programmed cell death protein 1;PD-L1, programmed cell death protein 1 ligand 1.

ICIs (CTLA-4, PD-1, and PD-L1 inhibitors) are used mainly for the treatment of solid and hematological tumors such as malignant melanoma, gastrointestinal tumors, non-small cell lung cancer, and renal cell carcinoma. Their main side effects include endocrine diseases such as hypothyroidism, hypophysitis, and adrenal insufficiency (5). ICI-related type I diabetes mellitus (T1DM) is a serious adverse effect of the use of these drugs, with incidences of 0.4–0.9% and 0.1–0.2% following the use of anti–PD-1 and anti–PD-L1 antibodies, respectively. T1DM caused by anti–CTLA-4 monotherapy is extremely rare and has been reported less frequently (6). As a glycoprotein on the surface of T cells, when combined with PD-L1 on the surface of tumor cells, PD-1 will affect the glucose uptake in T cells and make them lack the energy needed for activation (7) and the immune effect of the body will be damaged, and the tumor cells will escape. Therefore, toripalimab can restore the immune monitoring function of the body by inhibiting the PD-1/PD-L1 pathway to achieve the anti-tumor effect.Due to the inhibition of these pathways, β cells can not inhibit their own reactive CD8+T cells by up-regulating the expression of PD-L1, and the damage of β cells caused by de-inhibition is a possible pathogenesis of ICI-related diabetes caused by the use of toripalimab at present (8, 9)(**Figure 3**).

T1DM is characterized by absolute insulin deficiency secondary to T cell–mediated beta cell destruction and the presence of beta cell–related autoantibodies in serum (10). ICI-related diabetes also has these features, but can be distinguished from classic T1DM by its older age at onset (median, 65 years) (2), rapid onset of insulin deficiency, and lack of a “honeymoon period” (i.e., hidden onset with rapid development). The sudden loss of beta cell function distinguishes it from latent autoimmune diabetes in adults (11). Fulminant type 1 diabetes mellitus (FT1DM) is characterized by hyperglycemia, ketoacidosis, glycated hemoglobin (HbA1c) < 8.7%, elevated pancreatic enzyme levels, severe insulin deficiency (fasting C-peptide level < 0.3 ng/mL), and insulin autoantibody negativity. ICI-related diabetes of the fulminant type was first discovered and reported in Japan (12), and its onset is similar to that of conventional FT1DM, but with slower progression (13). Therefore, ICI-related diabetes has the characteristics of both classic type 1 diabetes and fulminant type 1 diabetes. At present, most literatures regard this type of diabetes as type 1 diabetes, but there is no report that it is classified as type 2 diabetes and other types of diabetes (14).

In case 1 presented here, the patient underwent surgery to remove a melanoma and received treatment with toripalimab, an anti–PD-1 antibody developed in China for the treatment of melanoma (15). The probability of hyperglycemia development was found to be much greater with toripalimab (55.6%) than with other ICIs, administered alone or in combination with chemotherapy, in an examination of 1063 cases (16). The combined administration of ICIs and chemotherapy may further increase the risk of adverse reactions such as hyperglycemia; the average onset time for this condition was about 2.7 drug cycles earlier with combined therapy than with monotherapy (after 4.5 cycles) in a systematic review of 90 cases (2). In addition, the onset of hyperglycemia is hidden in most patients undergoing toripalimab treatment, although the average blood glucose level in this group is 593 mg/L and the average glycosylated hemoglobin percentage is 7.5%, with a low or undetectable C-peptide level. The laboratory findings in case 1 presented here are basically consistent with this clinical picture.

Available evidence on the association of pancreatic autoantibodies with ICI-related diabetes is inconsistent. Some authors (17) have reported that most East Asian patients are negative for these autoantibodies, as in our case 1, whereas others have documented positivity for at least one pancreatic autoantibody, most commonly glutamic acid decarboxylase antibody, in 30–50% of cases (18). In addition, certain human leukocyte antigen (HLA) genotypes (e.g., the DR3-DQ2 and DR4-DQ8 haplotypes) are known to confer susceptibility to (fulminant) type 1 diabetes (19), and they (mainly DR4) have been detected in about 61% of patients with ICI-related diabetes (20). As the HLA genotype only partially explains some individuals’ increased risk of diabetes, we did not perform HLA genotyping in case 1.

In its 2018 guidelines, the American Society of Clinical Oncology recommended that a baseline serum glucose level be established before the initiation of ICI treatment, and that blood glucose measurement be performed every 12 weeks during the induction period and after each treatment cycle, and every 3–6 weeks thereafter (21). As ICI-induced diabetes may be mistaken for uncontrolled type 2 diabetes mellitus (T2DM), insulin antibody and C-peptide testing should be performed before treatment initiation for the risk stratification of patients with T2DM (22). For patients with suspected new or symptomatic diabetes, the timely monitoring of blood and urine ketones is necessary to prevent and identify DKA. Pancreatic damage caused by ICI use is irreversible, and insulin is the main means of treatment. As glucocorticoids have a toxic effect on pancreatic beta cells and may further increase the blood glucose level, they are not commonly used in the treatment of ICI-related diabetes (23).

Decisions about whether to discontinue ICI use should be made according to the severity of the clinical manifestations (21). In case 1 presented here, the patient’s blood glucose level did not decrease significantly after the cessation of anti–PD-1 treatment, and four insulin injections were administered.

## PI3Ki-related diabetes

The intracellular PI3K-AKT-mTOR signaling pathway is responsible for the regulation of various physiological functions, including the cell cycle, cell survival, protein synthesis, angiogenesis, and glucose metabolism (3). It is detected in nearly 44% of tumors, which makes it an excellent target for cancer therapy (24). Breast cancer, the malignancy with the highest incidence rate among women, shows changes in the PI3K pathway in about 58% of cases; the most common change is PIK3CA mutation, which activates the pathway (25).

**Figure 4:**
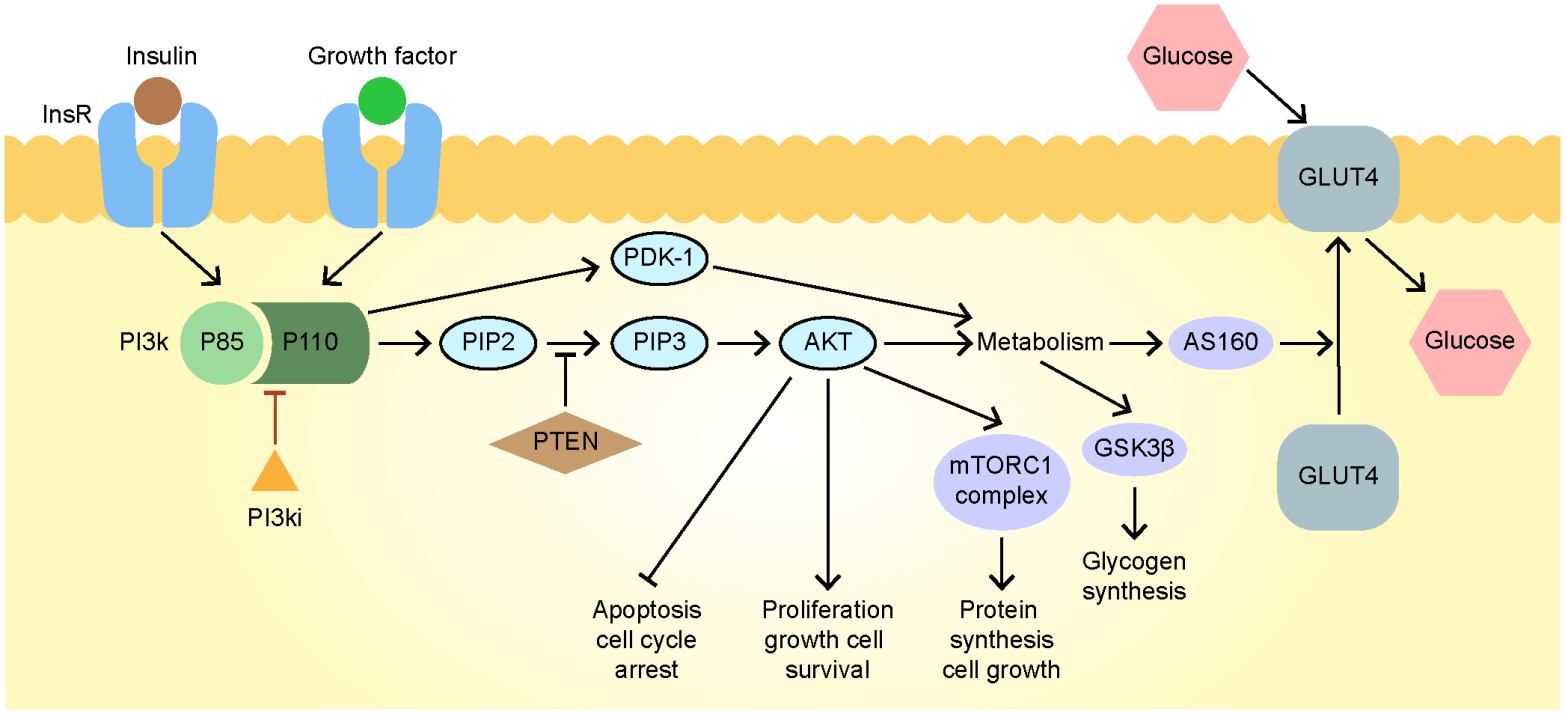
The mechanisms of drug action and hyperglycemia of alpelisib. AKT, protein kinase B; FOXO1, Fork head Box O1; GLUT4, glucose transporter type 4; GSK3, glycogen synthase kinase-3; Ins R, insulin receptor; mTORC1, mammalian target of rapamycin complex 1; PDK1, pyruvate dehydrogenase kinase; PI3K, phosphatidylinositol-3-kinase; PIP2, phosphatidylinositol 4, 5-bisphosphate; PIP3, phosphatidylinositol 3, 4, 5-trisphosphate; PTEN, phosphatase and tensin homolog.

PI3K has four subtypes (α, β, γ, and δ); the α subtype mediates most of the metabolic effects of the PI3K pathway, including insulin signaling in muscle, liver, and adipose tissues (26). The inhibition of the PI3K pathway blocks glucose uptake by skeletal and adipose tissues and promotes glycogen breakdown and liver gluconeogenesis, leading to hyperinsulinemia, hyperglycemia, and insulin resistance (27), is also the pathogenesis of type 2 diabetes (28). Although the SOLAR-1 study showed that the combination of alpelisib, an orally bioavailable PI3Kα-specific inhibitor blocking the The activation of PI3K by influencing the formation of heterodimer composed of the combination of p110 catalytic subunit and p85 regulatory subunit (29)(**Figure 4**), used to treat HR^+^, HER2^–^, PIK3CA-mutated advanced or metastatic breast cancer, and fulvestrant significantly improves progression-free survival in patients with such cancer, it also revealed that the incidence of hyperglycemia in these patients was up to 65% after medication use and that 6.3% of patients interrupted treatment due to poor blood sugar control (4).

PI3Ki-related diabetes caused by alpelisib has a median onset time of 15 days, with blood glucose spikes usually occurring 4–6 h after medication intake (4). This reaction, however, is reversible, and the blood glucose level can return to normal after alpelisib discontinuation. The probability of hyperglycemia is greater in patients aged ≥ 75 years with body mass indices ≥ 25 kg/m^2^ or HbA1c percentages ≥ 5.7%. The probability of DKA in patients with PI3Ki-related diabetes is 0.4%, much lower than that of ICI-related DKA (59–71.4%) (2, 30, 31), but this condition has a very poor prognosis and requires attention.

Cases of DKA in patients using alpelisib, identified from the PubMed database, are summarized in **Table 3**). The risk of alpelisib-induced DKA is increased and the onset of the condition is earlier in patients with long-term T2DM than in those without diabetes. Screening for diabetes risk factors before PI3Ki use and education in advance on diabetes symptoms and diagnostic criteria are recommended; PI3Ki treatment initiation is not recommended for patients with poorly controlled diabetes and those at high risk of developing hyperglycemia-related complications (27). Fasting blood glucose measurement once a week for 2 weeks before treatment initiation and monthly thereafter, and HbA1c monitoring every 3 months, are recommended. For patients with indications or symptoms of hyperglycemia, the monitoring frequency can be increased.

**Table 3:** Case reports of DKA with alpelisib use, retrieved from PubMed.

Authors	Age (y)	Pretreatment diabetes history	Time to onset	Blood glucose at onset (mg/dL)	KA treatment	Outcome
Leung et al. (32)	nd	T2DM	2 d	1137	nd	Stop alpelisib, empagliflozin
	58^1^	T2DM	10 d/2 h	707/nd	Insulin/insulin	Stop alpelisib and empagliflozin, increase gliclazide dosage/stop alpelisib
Thomas et al. (33)	76	T2DM	14 d	402	Alpelisib, metformin, glipizide	Stop alpelisib
Ekanayake et al (34).	37	GD	nd	397	Insulin, metformin, dulaglutide	Alpelisib, metformin, insulin
	44	No	300 d	nd	Insulin	Stop alpelisib, hypoglycemic drugs
Carrillo et al. (35)	66	No	14 d	300.6	Insulin, metformin, dapagliflozin	Stop alpelisib and insulin, switch to ixabepilone
Nguyen et al. (36)	73	No	11 d	>400	Insulin, metformin	Stop alpelisib and insulin, switch to vinorelbine
Fugere et al. (37)	48	No	26 d	302	Insulin	Stop alpelisib, insulin
Farah et al. (38)	49	No	60 d	387	Insulin	Alpelisib, metformin, insulin
Al Zeyoud et al. (39)	45	No	30 d	540	Insulin	Stop alpelisib

^1^Data on two DKA onsets are provided for this patient. DKA, diabetic ketoacidosis; T2DM, type 2 diabetes mellitus; nd, no data; GD, gestational diabetes.

First-line therapy for PI3Ki-related diabetes is administered with drugs that do not affect the PI3K pathway. The insulin sensitizer metformin can alleviate insulin resistance, inhibit liver gluconeogenesis and glycogen breakdown, and promote glucose uptake, but only after several weeks of use. Sodium-glucose cotransporter 2 (SGLT-2) inhibitors are recommended as second-line options and are often used in combination with metformin (27). In case 2 presented here, the patient was given alpelisib alone, alpelisib combined with insulin pump treatment, insulin pump treatment alone, alpelisib combined with metformin, and alpelisib combined with metformin and dapagliflozin, and her blood glucose level was monitored closely before and after meals to ensure the progression of the treatments’ hypoglycemic effect.

Notably, SGLT-2 inhibitors can cause DKA, characterized by mild insulin deficiency and insulin resistance (40), and the blood glucose level usually approaches normal or slight elevation (<288 mg/dL) when DKA occurs (41). As illustrated by a reported case of DKA in a patient taking taselisib (a PI3Ki) and canagliflozin (an SGLT-2 inhibitor) concurrently (42), the combined use of PI3Ki and SGLT-2 inhibitors should be undertaken with caution. A ketogenic diet can result in the consumption of liver glycogen stores, limitation of liver gluconeogenesis after PI3K pathway inhibition, reduction of the blood glucose and insulin levels, and increased efficacy of PI3Kis (43), but monitoring for DKA is still required.

## Conclusion

A large number of epidemiological studies have confirmed that both type 1 diabetes and type 2 diabetes have a certain risk of cancer (44).Studies have shown that the risk factors of cancer caused by diabetes include hyperglycemia, insulin resistance, hyperinsulinemia, insulin-like growth factor, obesity, inflammatory reaction and intestinal flora (45) and some cancers may increase the risk of diabetes. Although cancer treatment usually does not directly lead to diabetes, some therapeutic drugs and methods, such as radiotherapy, steroid hormones and some chemotherapy drugs, will lead to the increase of blood sugar level or inhibit the production of insulin, thus inducing diabetes (46, 47).

Here, we report on the diagnosis and treatment of ICI- and PI3Ki-related diabetes to improve medical workers’ awareness of these diseases. Screening for diabetes-related risk factors before cancer medication administration, close monitoring of the blood glucose level during medication use, and the prompt performance of relevant examinations and treatment initiation for patients who develop hyperglycemia or have clinical symptoms of diabetes are recommended to improve the prognosis.

## Data availability statement

The original contributions presented in the study are included in the article/supplementary material. Further inquiries can be directed to the corresponding author.

## Ethics statement

The studies involving humans were approved by Xiaogan Central Hospital Affiliated to Wuhan University of Science and Technology. The studies were conducted in accordance with the local legislation and institutional requirements. Written informed consent for participation was not required from the participants or the participants’ legal guardians/next of kin in accordance with the national legislation and institutional requirements. Written informed consent was obtained from the individual(s) for the publication of any potentially identifiable images or data included in this article.

## Author contributions

YG and MZ wrote the manuscript; YY conducted the design of the study and reviewed/edited the drafts, and is guarantor; LG, LL and CX collected and analyzed the data. All authors contributed to the article and approved the submitted version.
